# Filipino Children with High Usual Vitamin A Intakes and Exposure to Multiple Sources of Vitamin A Have Elevated Total Body Stores of Vitamin A But Do Not Show Clear Evidence of Vitamin A Toxicity

**DOI:** 10.1093/cdn/nzac115

**Published:** 2022-07-25

**Authors:** Reina Engle-Stone, Jody C Miller, Maria Fatima Dolly Reario, Charles D Arnold, Ame Stormer, Eleanore Lafuente, Anthony Oxley, Mario V Capanzana, Carl Vincent D Cabanilla, Jennifer Lynn Ford, Adam Clark, Thirumalaisamy P Velavan, Kenneth H Brown, Georg Lietz, Marjorie J Haskell

**Affiliations:** Institute for Global Nutrition, Department of Nutrition, University of California, Davis, Davis, CA, USA; Institute for Global Nutrition, Department of Nutrition, University of California, Davis, Davis, CA, USA; Helen Keller International, Malate, Manila, Philippines; Institute for Global Nutrition, Department of Nutrition, University of California, Davis, Davis, CA, USA; Helen Keller International, Malate, Manila, Philippines; Helen Keller International, Malate, Manila, Philippines; Human Nutrition Research Centre, Population Health Sciences Institute, Newcastle University, Newcastle Upon Tyne, United Kingdom; Food and Nutrition Research Institute, Department of Science and Technology, Bicutan, Taguig City, Philippines; Food and Nutrition Research Institute, Department of Science and Technology, Bicutan, Taguig City, Philippines; Department of Nutritional Sciences, College of Health and Human Development, The Pennsylvania State University, University Park, PA, USA; Human Nutrition Research Centre, Population Health Sciences Institute, Newcastle University, Newcastle Upon Tyne, United Kingdom; Institute of Tropical Medicine, Universitätsklinikum Tübingen, Tubingen, Germany; Vietnamese German Center for Medical Research (VG-CARE), Hanoi, Vietnam; Institute for Global Nutrition, Department of Nutrition, University of California, Davis, Davis, CA, USA; Human Nutrition Research Centre, Population Health Sciences Institute, Newcastle University, Newcastle Upon Tyne, United Kingdom; Institute for Global Nutrition, Department of Nutrition, University of California, Davis, Davis, CA, USA

**Keywords:** vitamin A, dietary intake, retinol isotope dilution, total body stores, toxicity, supplements, fortified foods, children, Philippines

## Abstract

**Background:**

Young children exposed to high-dose vitamin A supplements (VAS) and vitamin A (VA)–fortified foods may be at risk of high VA intake and high VA total body stores (TBS).

**Objectives:**

TBS and estimated liver VA concentration were compared among children with adequate or high VA intake and different timing of exposure to VAS, and associations between estimated liver VA concentrations and biomarkers of VA toxicity were examined.

**Methods:**

Children 12–18 mo of age (*n* = 123) were selected for 3 groups: *1*) retinol intake >600 µg/d and VAS within the past mo, *2*) retinol intake >600 µg/d and VAS in the past 3–6 mo, and *3*) VA intake 200–500 µg retinol activity equivalents (RAE)/d and VAS in the past 3–6 mo. Dietary intake data were collected to measure VA intakes from complementary foods, breast milk, and low-dose, over-the-counter supplements. TBS were assessed by retinol isotope dilution, and VA toxicity biomarkers were measured. Main outcomes were compared by group.

**Results:**

Mean (95% CI) VA intakes excluding VAS were 1184 (942, 1426), 980 (772, 1187), and 627 (530, 724) µg RAE/d, in groups 1–3, respectively; mean VA intake was higher in groups 1 and 2 compared with group 3 (*P* < 0.05). Geometric mean (GM) (95% CI) TBS were 589 (525, 661), 493 (435, 559), and 466 (411, 528) µmol, respectively. GM TBS and GM liver VA concentrations were higher in group 1 compared with group 3 (liver VA concentration: 1.62 vs. 1.33 µmol/g; *P* < 0.05). Plasma retinyl ester and 4-oxo-retinoic acid concentrations and serum markers of bone turnover and liver damage did not indicate VA toxicity.

**Conclusions:**

In this sample, most children had retinol intakes above the Tolerable Upper Intake Level (UL) and liver VA concentrations above the proposed cutoff for “hypervitaminosis A” (>1 µmol/g liver). There was no evidence of chronic VA toxicity, suggesting that the liver VA cutoff value should be re-evaluated. This trial was registered at www.clinicaltrials.gov as NCT03030339.

## Teaser

Young Filipino children exposed to multiple sources of dietary vitamin A, including supplements and fortified foods, have high vitamin A intake and body stores but no clear evidence of toxicity.

## Introduction

Vitamin A deficiency (VAD) has long been recognized as a public health problem contributing to child morbidity and mortality in many low- and middle-income countries (LMICs) (1). As vitamin A (VA) intervention programs have been scaled up globally in response to this challenge, concerns have been raised that children may be at risk of high VA intake and status because of exposure to multiple VA intervention programs, including market-driven food fortification (2, 3). In studies in Nicaragua (4) and Zambia (5), where children are exposed to VA-fortified sugar and VA supplements (VAS), stable isotope dilution methods were used to estimate total body VA stores (TBS) and liver VA concentrations in children in low-income communities; results indicated that ∼43% (Nicaragua; *n* = 21) and 59% (Zambia; *n* = 133) of study participants had liver VA concentrations above the proposed cutoff (>1 µmol/g liver) for “hypervitaminosis A” (6). However, the validity of the proposed cutoff value is uncertain because there are very limited data on the relation between liver VA concentrations and adverse health effects in humans (6, 7). In 1984, Olson (8) proposed a normal range for liver VA concentrations of 0.07 to 1.05 µmol/g. This was based on the hypothetical relation between serum and liver VA concentrations, which shows that serum VA concentrations are controlled homeostatically when liver VA concentrations are within this range, but increase markedly when the liver approaches saturation and is no longer able to store VA efficiently (7, 9). Later, Olson (9) changed liver VA status from “normal” to “excessive” for liver concentrations ranging from 0.7 to 1.05 µmol/g, and proposed a liver VA cutoff value of >1.05 µmol/g for “toxic” VA status. In 2010, the Biomarkers of Nutrition for Development (BOND) expert panel proposed a revised liver VA cutoff value of ∼10 µmol/g for VA toxicity, which was based on data from rhesus monkeys (10), and a cutoff value of >1 µmol/g for “subtoxic” status due to limited data on adverse effects (11). The term for the “subtoxic” category of status was changed to “hypervitaminotic” in 2016 (6).

To further explore the relation between exposure to multiple VA sources and intervention programs and VA status in children, the Global Vitamin A Safety Assessment (GloVitAS) Project was initiated in 2015. One aim of the GloVitAS project was to explore whether there is evidence of high VA intake or status among children in LMICs who are exposed to overlapping and/or prolonged VA interventions, and whether these are associated with biomarkers of VA toxicity. The Philippines was chosen as one of the GloVitAS study sites because several large-scale VA intervention programs are in place, and voluntarily fortified processed foods that are targeted to children are readily available. VAS are provided to Filipino children 6–59 mo of age per WHO recommendations [100,000 IU to children 6–11 mo of age; 200,000 IU to children 12–59 mo of age every 6 mo (12, 13)], and micronutrient powders (MNPs) that contain VA are potentially available at health centers and are targeted to infants and children 6–23 mo of age (14). Vitamin A fortification of wheat flour and cooking oil is mandatory, and >100 products in the marketplace are fortified with VA voluntarily by the food industry (15); these include VA-fortified “growing up” powdered milks, cereals, and snack foods that are targeted to young children. Over-the-counter, VA-containing supplements are also marketed for young children.

The aim of the study in the Philippines was to assess whether there was evidence of *1*) usual retinol intake above the Tolerable Upper Intake Level (UL); *2*) “hypervitaminosis A,” defined as a liver VA concentration >1 µmol/g based on the retinol isotope dilution (RID) method; and/or *3*) chronic VA toxicity based on serum markers of liver function, bone turnover, and/or VA metabolism among children who were exposed to multiple large-scale VA programs and identified as likely to have high intake. We assessed usual dietary VA intake, which included use of low-dose, over-the-counter supplements, and VA TBS in children who were selected for 3 groups: *1*) likely retinol intake >600 µg/d [the UL in North America (16) and the Philippines (17)] and VAS within the past 30 d, *2*) likely retinol intake >600 µg/d and VAS in the past 3–6 mo, and *3*) likely low/adequate VA intake [200–500 µg retinol activity equivalents (RAE)/d] and VAS in the past 3–6 mo. These groups distinguish between retinol and total VA intake because the UL for VA in North America and the Philippines is based on preformed retinol intake (micrograms per day); total VA intake includes both preformed retinol and provitamin A carotenoids (micrograms RAE per day). To determine group assignments, we developed a screening tool (18) to identify children likely to have “high intake” (retinol >600 µg/d) or “low/adequate-intake” (total VA intake of 200–500 µg RAE/d). We expected that mean usual retinol intake would exceed the UL of 600 µg/d in the high-intake groups and that mean dietary VA intake and TBS would be higher in children in the high-intake groups compared with the low/adequate-intake group. We also expected that any evidence of VA toxicity based on serum markers of bone turnover and liver damage would be observed in the high-intake groups, especially after recent VAS consumption, but not in the low/adequate-intake group.

## Methods

### Participants

The study was conducted in low- to middle-income, urban neighborhoods in Mandaluyong City in the National Capital Region of the Philippines. This area was selected because of high coverage (∼80%) by the national VAS program (19), participation in MNP distribution, and availability of VA-fortified foods in local markets. Children were eligible to participate in the study if they were 12–18 mo of age, their mothers were 18–49 y of age, and they and their mothers planned to stay in the study area for the duration of the study. Study procedures were described to mothers of potentially eligible children; those who were interested in participating provided written informed consent for themselves and their child. The study protocol was approved by the Research Ethics Board of the University of the Philippines-Manila, the Institutional Ethics Review Committee of the Food and Nutrition Research Institute in the Philippines, the Institutional Review Board of the University of California, Davis, and the Institutional Review Board of Newcastle University. The study was registered at ClinicalTrials.gov (NCT03030339).

Children were prescreened during the national VAS campaigns in March–May 2016 and September–November 2016 to determine whether they received VAS. Date of receipt of VAS was confirmed by checking the health information system that is maintained by health center staff, or the child's health card. Children who received VAS in the past 30 d or in the past 3–6 mo were eligible to participate in further screening.

Children and mothers were examined for physical signs and symptoms of VAD (Bitot's spots, conjunctival dryness, night blindness). Children were examined by a physician; maternal health was assessed by medical history. Children were weighed to the nearest 0.01 kg using an electronic scale (SECA 354; Seca), and length was measured to the nearest 0.1 cm using a stadiometer (SECA 217). Children were excluded from the study if the child or mother had chronic disease, signs or symptoms of VAD, if the child had a weight-for-length *z* score < –2 with respect to the age- and sex-specific WHO growth standards (20), or if the mother was breastfeeding >1 child.

As mentioned above, we developed a dietary screening tool to estimate VA intake in potentially eligible children (18). The tool was a semi-quantitative food-frequency questionnaire administered to mothers that consisted of questions about the child's consumption of breast milk, VA-fortified foods, selected natural sources of VA, and low-dose VA supplements (**Supplemental Methods**). The tool was pilot-tested in a different community in Manila and revised prior to the current study. A report comparing the results from the screening tool with those of the detailed dietary assessment is in preparation (18).

Children with an estimated retinol intake >600 µg/d were eligible for the high-intake groups if they had received VAS in the past month (group 1) or in the past 3–6 mo (group 2). Children with an estimated VA intake between 200 and 500 µg RAE/d who received VAS in the past 3–6 mo were eligible for the low/adequate-intake group (group 3). Note that none of the children received VAS during the 28-d study period; they received VAS either 30 d prior to enrollment (group 1) or 3–6 mo prior to enrollment (groups 2 and 3). Children were treated presumptively for intestinal helminths (200 mg albendazole) during the screening visit.

### Overview of study design and objectives

We used a 28-d population kinetics (“super-child”) study design and compartmental modeling to determine retinol kinetic parameters in children and develop population-specific coefficients for use in the RID equation, which we used to estimate TBS in individual children, as described in detail previously (21–22). The 28-d super-child kinetic studies were conducted separately for each of the 3 groups of children (Supplemental Methods). A timeline for procedures within each super-child study is summarized in **Table 1**. Briefly, during the 28-d study period, dietary VA intake was assessed in children using a combination of methods, and TBS were estimated using the RID equation at 4 d after tracer administration. We chose to determine TBS at 4 d, as this was shown to provide reasonably accurate predictions of TBS for individual children in a study of theoretical children with a wide range of assigned TBS values [i.e., 66–80% of theoretical individual children had RID-predicted TBS within 25% of their assigned values in 5 different scenarios; see reference (23) for details]. The specific aims of the study were as follows: *1*) to assess whether mean retinol intake in the high-intake groups exceeded the UL of 600 µg/d, *2*) to compare mean total VA intake and prevalence of inadequate and high VA intakes among the 3 groups, *3*) to compare mean indicators of VA status [TBS, estimated liver VA concentration, and plasma retinol and serum retinol-binding protein (RBP) concentrations] among the 3 groups, and *4*) to compare mean values for biomarkers of VA toxicity among the 3 groups [i.e., plasma concentrations of retinyl esters and 4-oxo-retinoic acid, serum markers of bone resorption [serum tartrate-resistant acid phosphatase 5b (TRACP 5b)] and bone formation [procollagen type 1 N-terminal propeptide (PINP)] and serum markers of liver damage [alanine-transaminase (ALT) activity; aspartate-transaminase (AST) activity]. We assessed inflammation and iron status on study day 4, and determined the relation between usual total VA intake and TBS. We also examined associations among dietary VA intake and indicators of VA status, inflammation, and VA toxicity.

**TABLE 1:** Summary of data collection during 28-d population kinetics (super-child) studies^1^

Study day	Child	Mother
Day 0	Morbidity assessmentHeel-prick for measurement of Hb and CRP[^13^C_10_]-retinyl acetate dosing30-d supplement use/liver consumption questionnaire	−
Day 4	Blood collection for estimation of TBS^2^ Morbidity assessment	−
Randomly assigned sampling time (6, 9, 12 h; 1, 2, 7, 11, 16, 22, or 28 d after [^13^C_10_]-retinyl acetate dosing)	Blood collection for construction of “super-child” plasma retinol response curve Morbidity assessment	−
Days 6–20	Saliva collection for estimation of breast-milk intake	Administration of deuterium oxide (day 6) and saliva collection for estimation of breast-milk intake
Day 20		Breast-milk collection for measurement of milk vitamin A
Days 0–28	∼4 × 24-h recalls, and a 12-h in-home food intake observation + 12-h recall observation	2 × 24-h recalls^3^

1 AGP, α_1_-acid glycoprotein; CRP, C-reactive protein; RBP, retinol-binding protein; RID, retinol isotope dilution; sTfR, soluble transferrin receptor; TBS, total body stores of vitamin A.

2 TBS was assessed using the RID method; other measurements included plasma retinol concentration and serum concentrations of RBP, CRP, AGP, ferritin, and sTfR.

3 Data not shown; to be presented separately.

### Administration of tracer and super-child study design

On day 0, children were screened for anemia and high C-reactive protein (CRP) using a point-of-care device (Quik-Read Go wrCRP+ Hb; Aidian Oy). Children with hemoglobin >100 g/L and CRP ≤5 mg/L received an oral dose of [^13^C_10_]-retinyl acetate (1.168 µmol as [^13^C_10_]-retinol; 99.6% all*-trans* purity; certified fit for human consumption by Food Chemical Codex testing; Buchem BV) dissolved in sunflower oil (see Supplemental Methods for details). The dose was administered directly into the child's mouth using a positive displacement pipette (Microman E M250E; Gilson, Inc.); then, children were given high-fat cookies to enhance absorption of the dose. They were scheduled to return to the study clinic for blood collection 4 d after administration of the dose and at 1 of 10 additional randomly assigned time points (6, 9, 12 h; or 1, 2, 7, 11, 16, 22, 28 d after dosing) for the super-child kinetic study; thus, there were approximately 5 children per time point in each of the 3 groups (23).

### Assessment of dietary VA intake

On day 0, a questionnaire was administered to mothers to obtain information on the child's use of MNPs and over-the-counter VA-containing supplements in the past 30 d. During each 28-d super-child study, four to five 24-h diet recalls were scheduled on nonconsecutive days for trained interviewers to obtain information on the child's food intake. Recall interviews used a multiple-pass method and included collection of recipe data for any recipes prepared in the home, including the amounts of water used to prepare powdered milk (24). In addition, one 12-h in-home weighed food record with 12-h recall was conducted for each child, with the following exceptions: *1*) for group 2, inadvertently, the 12 h recall was not conducted, and *2*) for 25 children, the 12-h observation and 12-h recall could not be conducted due to security concerns; for these latter cases, where possible, interviewers administered an additional 24-h recall instead. We used the Nutrient Data System for Research (Nutrition Coordinating Center, University of Minnesota, USA) as the base for the food-composition table, supplemented with information from the database of the Food and Nutrition Research Institute (FNRI) in the Philippines (25). (See Supplemental Methods and **Supplemental Results** for details on estimation of VA content of breast milk, fortified foods, and supplements.)

### Estimation of usual dietary intake

We used the National Cancer Institute (NCI) method to estimate usual energy and nutrient intake distributions for daily intake measured by 24-h recalls and 12-h observations with 12-h recalls (26). We then used the “shrink then add” approach (27) to include the contribution of over-the-counter supplements (30-d questionnaire) and estimated intake from breast milk. (See Supplemental Methods for more details.)

### Child morbidity

Data on symptoms of selected illnesses were collected for each child by administering a questionnaire to mothers (see Supplemental Methods).

### Blood and breast-milk collection and processing

Venous blood (6 mL) was collected from children by a trained phlebotomist; plasma (EDTA-treated) and serum were obtained by centrifugation (1500 × *g*, for 15 min at room temperature) and transferred into cryovials. Breast milk (∼15 mL) was collected from mothers by manual expression or by using a breast pump depending on personal preference on day 20. Fat was measured in fresh milk in triplicate using the creamatocrit method (Creamatocrit Plus Centrifuge; EKF Diagnostics). Remaining milk was mixed well and aliquoted into cryovials for later analysis of the VA content. Biological samples were stored in a cooler on ice packs until being transferred on the day of collection to the FNRI, where they were stored at –80°C. All procedures were carried out in dim light.

### Analytical procedures

Plasma and serum samples were shipped in dry ice to Newcastle University (Newcastle Upon Tyne, UK). Plasma (400 µL) was extracted [modified from Aebischer et al. (28)] for retinol and retinyl esters, and 200 µL plasma was extracted [modified from Barua (29)] for more polar retinoids, such as 4-oxo-retinoic acid. Each extracted residue was reconstituted in 100 µL ethanol. Ten or 20 µL of each extract was analyzed by LC-MS/MS for [^12^C]- and [^13^C_10_]-retinol and retinyl esters (30), with quantitation of 4-oxo-retinoic acid at *m/z* 315→159. The applied method did not separate all-*trans*- from 13-*cis*-4-oxo-retinoic acid; the data presented are the sum of both isomers. The intra-day and inter-day CVs for the retinol measurements were 2.3% and 4.4%, respectively. (See Supplemental Methods for details on analyses of breast-milk VA; serum indicators of VA and iron status; and serum markers of inflammation, bone turnover, liver function and hepatitis.)

### Prediction of VA total body stores

We used the RID equation (Equation 1), previously presented by Green et al. (21), to estimate TBS in individual children 4 d after ingestion of the [^13^C_10_]-retinyl acetate dose:

(1) $$\begin{eqnarray*}
{\rm{TBS }} = Fa \times S \times 1/{\rm{S}}{{\rm{A}}}_{\rm{p}}
\end{eqnarray*}$$

where *Fa* is the fraction of the oral tracer dose absorbed and retained in stores at 4 d, *S* is retinol specific activity in plasma/stores at 4 d, and SA_p_ is retinol specific activity in plasma [fraction of dose in plasma (FD_p_)/total retinol in plasma (µmol)] at 4 d. A value of 2.44 was used for individuals in each group for the composite coefficient *Fa* × *S*; that value was derived by applying compartmental modeling to the super-child dataset [geometric mean (GM) plasma retinol fraction of dose vs. time] generated for all participants in this study, as described previously (22). Liver VA concentration was calculated for each child using body surface area to estimate liver weight (31) and assuming 80% of TBS are present in the liver (32).

### Sample size

The sample size was based on detection of mean retinol intake >600 µg/d in the high-intake groups. Because there were no data on VA intake for children in our study area, we estimated VA intake for this purpose as follows: for children in the high-intake groups we assumed that mean VA intake from breast milk would be ∼419 µg/d [breast-milk VA of 2.75 µmol/L (33) and breast-milk intake of 0.533 L/d (34)] and that retinol intake from complementary foods (including fortified foods) would be ∼300 µg/d for a total VA intake of 719 µg/d. We assumed an SD of ∼31%, which is based on the variability in breast-milk intake among Malawian infants 9–10 mo of age (35), because we did not have data on variability in VA intake from breast milk or complementary foods for children in our study area. This resulted in an estimated mean ± SD retinol intake of ∼719 ± 223 µg/d. Under these assumptions, 40 children per group would be needed to detect mean retinol intake >600 µg/d with 80% power and ɑ = 0.05. To account for attrition, and because we expected the variability in VA intake to be greater when intakes from non–breast-milk foods were included, we increased this number by ∼25% to 50 per group (*n* = 150 total). This sample size would be sufficient to include 5 children at each of the 10 assigned super-child time points in all 3 groups (*n* = 150 total) (23). Assuming a mean liver VA concentration of 1 µmol/g in the high-intake groups (6), a CV of 56% for the mean liver VA concentration (36), and an effect size of at least 0.5, this would allow us to detect a 29% difference in mean liver VA concentrations between the high-intake and low/adequate-intake groups.

### Statistical analysis

Descriptive statistics were calculated for all variables and residual distributions were assessed for normality by visual examination of histograms and by the Shapiro–Wilk “W” test. Skewed distributions were natural-log transformed and reported as GMs and 95% CIs. At enrollment, participant characteristics were compared among groups using ANOVA for normally distributed variables and logistic regression for binary outcomes, each with post hoc Tukey–Kramer pairwise comparisons.

Usual dietary intakes were estimated by the NCI method, as described above. VA intake was considered to be inadequate if it was below the North American Estimated Average Requirement [EAR; 210 µg RAE/d; (16)]. VA intake was considered to be high if preformed retinol intake was above the North American and Philippines’ UL of 600 µg retinol/d (16, 17) or above the European UL of 800 µg preformed VA (retinol and retinyl esters)/d (37). The prevalence of low and high VA intakes was based on these cutoffs. Bootstrapped SEs were used to construct 95% CIs and conduct significance testing based on unequal variance *t*-testing. As prespecified in our data analysis plan, we first tested for differences between groups 1 and 2, and if they did not differ, we pooled the groups for testing against group 3. Group comparisons were controlled for child age by fixing child age at the sample mean.

Indicators of VA status, VA toxicity, and inflammation were compared among groups using ANCOVA with Tukey–Kramer pairwise comparisons. These analyses were conducted with and without the inclusion of covariates. Potential covariates were selected based on biological relevance and were prespecified in our analysis plan [child age, sex, body weight, length, BMI, % of days with morbidity symptoms, serum concentrations of CRP, α_1_-glycoprotein (AGP), ferritin, soluble transferrin receptor (sTfR), hemoglobin concentration, breastfeeding status, and timing of VAS receipt]. The prevalences of binary indicators were similarly compared by group using logistic regression. Because RBP is a negative acute-phase reactant, plasma/serum concentrations of retinol and RBP decrease during inflammation. For this reason, plasma retinol and serum RBP concentrations were adjusted for inflammation using the Biomarkers Reflecting Inflammation and Nutritional Determinants of Anemia (BRINDA) method, which is a linear regression approach for adjusting plasma/serum concentrations of retinol and RBP for serum markers of inflammation (CRP and AGP) (38). Both inflammation-adjusted and non–inflammation-adjusted results are presented. Plasma retinol concentration <0.7 µmol/L was used to indicate VAD. A population-specific cutoff value for serum RBP that is analogous to the cutoff of <0.7 µmol/L for plasma retinol concentration was determined following the Liu optimal cutpoint determination method (39). Serum concentrations of CRP >5 mg/L and serum concentrations of AGP >1 g/L were used to indicate inflammation. The proposed cutoff of >1 µmol/g liver for “hypervitaminosis A” (6) was used to indicate high VA status. A cutoff value for TBS has not been established; mean TBS values were compared by group as described above.

Spearman correlations were used to examine relations among indicators of VA status and between indicators of inflammation and VA status. Regression analysis was used to examine associations between biomarkers of VA toxicity and estimated liver VA concentration, and biomarkers of VA toxicity and usual dietary VA intake. Regression analysis was also used to examine the relation between predicted VA intake (estimated from the NCI indivint macro, as described above) and estimated liver VA concentration, in groups 2 and 3 combined only, because children in those groups received VAS at least 90 d prior to RID testing, whereas children in group 1 received VAS in the past 30 d. These analyses were conducted with and without potential covariates that were selected based on prespecified biological relevance (child age, child sex, child weight, breastfeeding status, serum concentrations of CRP and AGP).

Recent studies suggest that later blood sampling times (12–28 d after dosing) may provide more accurate estimates of TBS (40, 41). For this reason, we compared RID-predicted TBS at 4 d versus 16–28 d after dosing among children who had blood collected at both the early and later time points (*n* = 29). We also examined relations between biomarkers of VA toxicity and estimated liver VA concentration by quintile, in addition to the individual-level analyses (see Supplemental Methods for more details).

## Results

### Participant and household characteristics at enrollment

**FIGURE 1:**
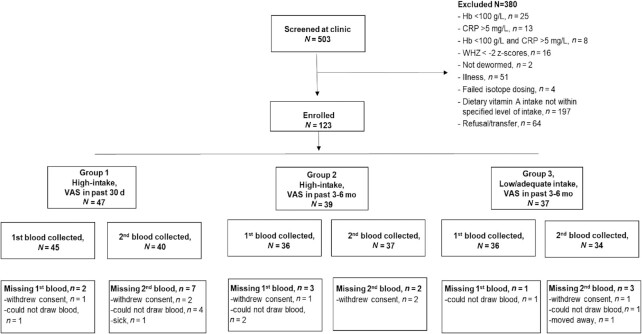
Enrollment and losses to follow-up of children by study group. CRP, C-reactive protein; Hb, hemoglobin; VAS, vitamin A supplements; WHZ, weight-for-height *z* score.

A total of 503 children were screened for eligibility and 123 children were enrolled in the study (**Figure 1**). The main reason for exclusion was that usual VA intakes were not in the specified ranges. Children were 13.9 mo of age, on average, and children in group 3 were slightly younger than those in groups 1 and 2 (**Table 2**). Mean length was lower in group 3 than in group 2; however, there was no difference in length-for-age *z* scores by group. Overall, approximately 51% of children were breastfed; the percentage of breastfed children was higher in group 3 than in groups 1 and 2 (Table 2). Maternal and household characteristics are shown in **Table 3**.

**TABLE 2:** Child characteristics by group at enrollment^1^

	Group 1 (high-intake + VAS in past 30 d)	Group 2 (high-intake + VAS in past 3–6 mo)	Group 3 (low/adequate-intake + VAS in past 3–6 mo)
*n*	47	39	37
Age, mo	14.0 (13.5, 14.4)^a^	15.1 (14.5, 15.6)^b^	12.6 (11.7, 13.4)^c^
Male sex, *n* (%)	27 (61.4)	20 (54.1)	16 (43.2)
Length, cm	73.6 (72.7, 74.6)^a,b^	74.8 (73.8, 75.8)^a^	72.4 (71.3, 73.4)^b^
Length-for-age *z*-score < −2 (%)	25.0 (11.7, 38.3)	24.3 (9.8, 38.8)	27.0 (12.0, 42.0)
Weight, kg	8.9 (8.5, 9.2)	9.0 (8.6, 9.4)	8.5 (8.1, 8.8)
Weight-for-age *z*-score < −2 (%)	15.9 (4.7, 27.2)	13.5 (2, 25.1)	13.5 (2, 25.1)
Weight-for-length *z*-score	−0.4 (−0.7, 0.0)	−0.4 (−0.7, −0.1)	−0.5 (−0.7, −0.2)
Hemoglobin, g/L	11.8 (11.5, 12.1)	11.8 (11.5, 12.2)	11.5 (11.2, 11.8)
Currently breastfed, *n* (%)	22 (46.8)^a^	14 (35.9)^a^	26 (70.3)^b^

1 Values are mean (95% CI) or *n* (%). Superscripts letters indicate Tukey–Kramer pairwise testing *P* < 0.05. Note: weight-for-length *z-*score is presented as the mean (95% CI) because children with values ←2 *z* scores were excluded from the study. VAS, high-dose vitamin A supplement.

**TABLE 3:** Characteristics of households by group at enrollment^1^

	Group 1 (high-intake + VAS in past 30 d)	Group 2 (high-intake + VAS in past 3–6 mo)	Group 3 (low/adequate-intake + VAS in past 3–6 mo)
*n*	44	38	34
Household size, *n*	6.3 (5.3, 7.2)	6.4 (5.5, 7.4)	5.9 (5.2, 6.5)
Family-owned assets			
Home	29 (65.9%)	23 (60.5%)	24 (70.6%)
Refrigerator and/or freezer	16 (36.4%)	12 (31.6%)	12 (35.3%)
Television	40 (90.9%)	29 (76.3%)	29 (85.3%)
Bicycle	10 (22.7%)	16 (42.1%)	10 (29.4%)
Motorcycle	13 (29.5%)	6 (15.8%)	6 (17.6%)
Access to electricity	41 (93.2%)	37 (97.4%)	34 (100%)
Water source^2^			
Piped water	21 (47.7%)	26 (68.4%)	19 (55.9%)
Water refilling station	25 (56.8%)	16 (42.1%)	22 (64.7%)
Other	1 (2.3%)	3 (7.9%)	1 (2.9%)
Head of household literacy	44 (100%)	38 (100%)	34 (100%)
Maternal age, y	29.0 (26.9, 31.0)	28.1 (25.9, 30.4)	26.9 (24.6, 29.2)
Marital status^3^			
Married	15 (34.1%)	11 (28.9%)	12 (35.3%)
Single	6 (13.6%)	6 (15.8%)	3 (8.8%)
Unmarried, living with partner	22 (50%)	21 (55.3%)	18 (52.9%)
Number of live births	2.4 (2, 2.9)	2.6 (2.1, 3.2)	2.4 (1.9, 2.9)
Literacy	44 (100%)	38 (100%)	34 (100%)
Maternal educational level attained			
Grade 4–6	4 (9.1%)	4 (10.5%)	3 (8.8%)
Some secondary schooling	6 (13.6%)	6 (15.8%)	4 (11.8%)
High school graduate	18 (40.9%)	10 (26.3%)	13 (38.2%)
Vocational/college	16 (36.6%)	17 (44.7%)	13 (38.2%)
Maternal occupation:			
Housewife	29 (65.9%)	25 (65.8%)	26 (76.5%)
Other^4^	15 (34.1%)	13 (34.2%)	8 (23.5%)

1 Values are geometric means (95% CI) or *n* (%).VAS, high-dose vitamin A supplement.

2 Some women in each group reported more than one water source.

^3^One woman in group 1 and one woman in group 3 were separated from their partners.

4 “Other” included shop/market worker, housemaid, technical/skilled factory worker, small business owner, and “other.”

### Usual dietary intake of energy, fat, and VA

Mean usual energy intake ranged from 791 to 925 kcal/d among the 3 groups (**Table 4**). Because there were no differences in mean intakes of energy, fat, VA, or retinol between the 2 high-intake groups, these groups were combined and compared with the low/adequate-intake group. Mean usual intakes of energy were higher in groups 1 and 2 combined (high-intake groups) than in group 3 (low/adequate-intake group), consistent with the younger age in group 3. Mean usual intakes of VA and retinol were also higher in the combined high-intake groups than in the low/adequate-intake group. The mean (SD) intake of VA, expressed per 1000 kcal and including all sources of VA, was 1288 (643) µg RAE/1000 kcal in group 1, 1037 (640) in group 2, and 769 (385) in group 3. The mean (SD) intake of VA from food sources was 515 (259) µg RAE/1000 kcal in group 1, 619 (224) in group 2, and 356 (230) in group 3. In all groups, retinol accounted for ≥97% of total VA intake. The lower 95% confidence limit for mean retinol intake in both of the high-intake groups was above the North American UL of 600 µg/d and above the European Union (EU) UL of 800 µg/d in group 1 but not in group 2. The lower 95% confidence limit for mean retinol intake in the low/adequate-intake group was below the North American UL but the upper limit of the CI exceeded 600 µg/d. The prevalence of inadequate VA intake was low overall: 3.2% in the low/adequate-intake group and 1.5% in the combined high-intake groups (Table 4). The prevalence of high retinol intakes was greater in the combined high-intake groups than in the low/adequate-intake group, based on the North American UL of >600 µg/d (79.9% vs. 40.3%) and the EU UL of >800 µg/d (59.0% vs. 20.4%).

**TABLE 4:** Usual dietary intake of energy, fat, and vitamin A in children by group^1^

	Group 1 (high-intake + VAS in past 30 d)	Group 2 (high-intake + VAS in past 3–6 mo)	Group 3 (low/adequate-intake + VAS in past 3–6 mo)
*n*	47	39	37
Energy intake, kcal/d	889 (784, 994)	925 (818, 1033)	791 (706, 875)
Fat intake, g/d	34 (30, 37)	33 (29, 37)	31 (27, 34)
Vitamin A intake, µg RAE/d	1184 (942, 1426)^a^	980 (772, 1187)^a^	627 (530, 724)^b^
Vitamin A intake <210 µg RAE/d, %	2.2 (0.0, 5.2)	0.8 (0.0, 2.5)	3.2 (0.0, 7.6)
Retinol intake, µg/d	1169 (925, 1412)^a^	949 (742, 1155)^a^	612 (515, 709)^b^
Retinol intake >600 µg/d, %	84.5 (75.9, 93.1)^a^	75.3 (62.4, 88.1)^a^	40.3 (27.2, 53.4)^b^
Retinol intake >800 µg/d, %	65.7 (52.0, 79.3)^a^	52.3 (37.2, 67.3)^a^	20.4 (9.2, 31.6)^b^

1 Values are mean or prevalence with bootstrapped 95% CIs. Superscript letters indicate *P* < 0.05 testing differences between group 3 and groups 1 and 2 combined, controlling for child age (i.e., child age fixed at sample mean). RAE, retinol activity equivalent; VAS, high-dose vitamin A supplement.

### Sources of dietary VA

Based on 24-h recall data, complementary foods provided 48% to 69% of total VA intake among the 3 groups and fortified powdered milk accounted for 56% to 78% of VA intake from complementary foods (**Supplemental Table 1**). Over-the-counter, low-dose VA-containing supplements provided 18% to 41% of total VA intake, and breast milk provided 7% to 30%. Only 1 child was reportedly consuming MNP, which was not available at the health centers during the study period; this child's results were included in the analysis.

### Child morbidity, inflammation, and iron status indicators

The overall mean percentage of days with reported symptoms of illness was low (cough, 9.7%; nasal discharge, 13.1%; fever, 2.4%; and diarrhea, 1.8%); the mean percentage of days with nasal discharge or purulent nasal discharge differed by group (**Supplemental Table 2** and Supplemental Results).

On study day 4, ∼30% of children had inflammation (CRP >5 mg/L and/or AGP >1g/L) and ∼23% were iron deficient (inflammation-adjusted serum ferritin <12 µg/L). Prevalences of inflammation and iron deficiency did not differ by group (**Supplemental Table 3**).

### VA status indicators

GM plasma retinol concentration was higher in group 2 (high VA intake; VAS in past 3–6 mo) than in group 3 (low/adequate VA intake; VAS in past 3–6 mo); however, after correcting for inflammation, GM plasma retinol concentration did not differ by group (**Table 5**). GM serum RBP concentrations did not differ by group before or after correcting for inflammation. The overall prevalence of low inflammation-adjusted plasma retinol (<0.7 µmol/L) and serum RBP concentrations [<0.78 µmol/L (population-specific cutoff value)] was low and did not differ by group (Table 5).

**TABLE 5:** Vitamin A status indicators on study day 4 by study group^1^

	Group 1 (high-intake + VAS in past 30 d)	Group 2 (high-intake + VAS in past 3–6 mo)	Group 3 (low/adequate-intake + VAS in past 3–6 mo)
*n*	41	35	35
Plasma retinol, µmol/L	0.98 (0.92, 1.05)^a,b^	1.12 (1.04, 1.21)^a^	0.88 (0.82, 0.95)^b^
Inflammation-adjusted plasma retinol, µmol/L	1.11 (1.04, 1.18)	1.21 (1.13, 1.29)	1.06 (0.99, 1.13)
Serum RBP, µmol/L	1.11 (1.02, 1.20)	1.13 (1.03, 1.24)	0.94 (0.86, 1.03)
Inflammation-adjusted serum RBP, µmol/L	1.25 (1.15, 1.35)	1.22 (1.12, 1.33)	1.13 (1.04, 1.23)
Plasma retinol <0.7 µmol/L, %	4.7	0	11.4
Inflammation-adjusted plasma retinol <0.7 µmol/L, %	0	0	0
Serum RBP <0.78 µmol/L, %	9.3	8.3	25.7
Inflammation-adjusted serum RBP <0.78 µmol/L, %	2.3	8.3	2.9
TBS,^2^ µmol	589 (525, 661)^a^	493 (435, 559)^a,b^	466 (411, 528)^b^
Liver vitamin A concentration,^2^ µmol/g	1.62 (1.45, 1.81)^a^	1.34 (1.19, 1.51)^a,b^	1.33 (1.18, 1.50)^b^

1 Values are geometric mean (95% CI) or prevalences. Superscript letters indicate Tukey–Kramer pairwise testing *P* < 0.05 controlling for child age. BRINDA, Biomarkers Reflecting Inflammation and Nutritional Determinants of Anemia; RBP, retinol-binding protein; SA_p_, retinol specific activity in plasma; TBS, total body stores of vitamin A; VAS, high-dose vitamin A supplement.

2 One child in group 1 had an SA_p_ value ∼10 times greater than the mean value for the group and was excluded from the analysis. Plasma retinol and serum RBP concentrations were adjusted for inflammation using the BRINDA method (38).

**FIGURE 2:**
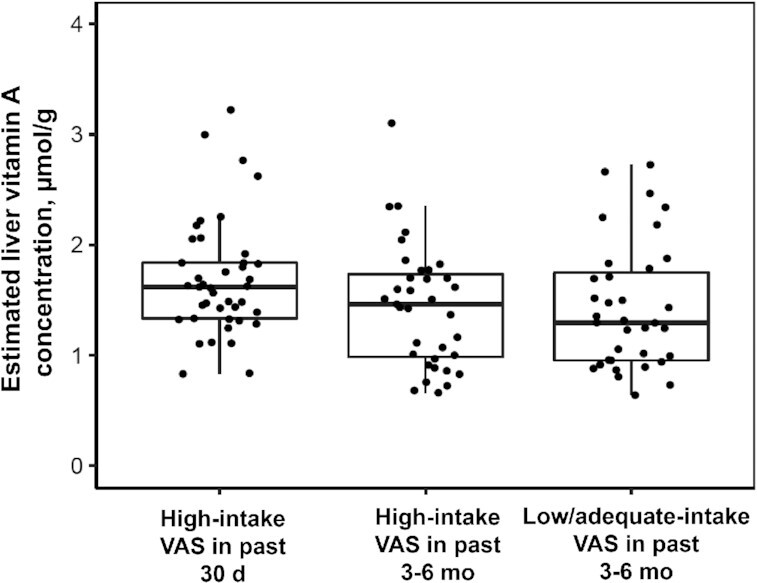
Distribution of liver vitamin A concentrations by study group. Box plots (p25, median, p75) are shown for each group. Dots are individual data points. GM (95% CI) liver vitamin A concentration differed between groups 1 and 3 (*P* < 0.05) controlling for child age. GM, geometric mean; p25, 25th percentile; p75, 75th percentile; VAS, high-dose vitamin A supplement.

Overall, individual TBS ranged from 211 to 1208 µmol. GM TBS were higher in group 1 (high VA intake; VAS in past 30 d) than in group 3 (low/adequate intake; VAS in past 3–6 mo) (Table 5). GM TBS did not differ between groups 1 and 2 (high-intake groups) or between groups 2 (high VA intake; VAS in past 3–6 mo) and 3 (low/adequate intake; VAS in past 3–6 mo). Estimated individual liver VA concentration ranged from 0.64 to 3.22 µmol/g. GM estimated liver VA concentration was higher in group 1 (high VA intake; VAS in past 30 d) than in group 3 (low/adequate intake; VAS in past 3–6 mo), and there were no other differences by group. Notably, the vast majority of children (78.4%, including 69% in group 3) had estimated liver VA concentrations >1 µmol/g, which has been proposed to suggest “hypervitaminosis A” (6). One child each in groups 1 and 2 (high-intake) had an estimated liver VA concentration >3 µmol/g; no children in group 3 had a liver VA concentration >3 µmol/g (**Figure 2**). There was no association between estimated usual total VA intake and estimated liver VA concentration (or TBS) in the groups that received the VAS 3–6 mo before commencing the study.

Correlations among indicators of VA status and inflammation showed that TBS, liver VA concentration. and retinol specific activity in plasma (SA_p_) were not correlated with inflammation markers, whereas plasma retinol concentration and fraction of dose in plasma (FD_p_) were negatively correlated with inflammation markers. Serum RBP concentration was also negatively correlated with serum CRP concentration (**Supplemental Table 4**).

### RID-predicted TBS in the same children at 4 d compared with 16–28 d after dosing with labeled VA

RID-predicted TBS at 4 d after dosing were highly correlated with data obtained from the same individuals at 16–28 d after dosing (Spearman's correlation: *r* = 0.78; *P* < 0.0001; *n* = 29; **Supplemental Figure 1**; Spearman's rank order correlation: *r* = 0.77; *P* < 0.0001; *n* = 29 **Supplemental Figure 2**).

### Plasma/serum markers of VA toxicity

The mean nonfasting plasma retinyl ester concentration ranged from 0.06 to 0.12 µmol/L and was higher in group 2 (high VA intake; VAS past 3–6 mo) than in groups 1 (high VA intake; VAS in past 30 d) and 3 (low/adequate intake; VAS in past 3–6 mo) (**Table 6**). One child in group 2 had a plasma retinyl ester concentration (0.646 µmol/L) above the proposed cutoff value of ≥0.380 µmol/L (42), but no other children exceeded this concentration. Mean plasma 4-oxo-retinoic acid concentrations did not differ by group.

**TABLE 6:** Plasma retinyl esters, plasma 4-oxo-retinoic acid, and serum markers of bone metabolism and liver damage by study group^1^

	Group 1 (high-intake + VAS in past 30 d)	Group 2 (high-intake + VAS in past 3–6 mo)	Group 3 (low/adequate-intake + VAS in past 3–6 mo)
Retinyl esters, µmol/L	0.08 (0.07, 0.10)^a^	0.12 (0.10, 0.14)^b^	0.06 (0.04, 0.08)^a^
4-oxo-retinoic acid, nmol/L	6.42 (5.06, 7.78)	6.43 (4.71, 8.15)	6.47 (4.78, 6.16)
TRACP 5b, U/L	8.4 (7.7, 9.0)^a^	10.4 (9.5, 11.2)^b^	10.2 (9.3, 11.1)^b^
PINP, µg/L	1186 (1075,1297)	1099 (965,1232)	1281 (1143,1419)
ALT, U/L	17.2 (15.3, 19.0)	13.9 (11.7, 16.2)	17.2 (14.6, 19.7)
AST, U/L	40.4 (37.8, 43.0)	38.5 (35.3, 41.6)	37.9 (34.3, 41.5)
AST/ALT	2.68 (2.43, 2.94)^a^	2.93 (2.62, 3.24)^a^	2.12 (1.76, 2.47)^b^

1 Values are means (95% CI) or prevalences (95% CI). Superscript letters indicate Tukey–Kramer pairwise testing *P* < 0.05 controlling for child age, serum CRP, serum AGP, length, BMI, child sex, and breastfeeding status. Sample size differs by outcome and is as follows by group: serum TRACP 5b (38, 27, 26), serum PINP (39, 32, 32), ALT (30, 26, 22), serum AST (27, 22, 18), serum AST/ALT (27, 22, 18), plasma retinyl esters (43, 36, 35), plasma 4-oxo-retinoic acid (39, 30, 31). AGP, α_1_-glycoprotein; ALT, alanine aminotransferase; AST, aspartate aminotransferase; CRP, C-reactive protein; PINP, procollagen I intact N-terminal; TRACP 5b, tartrate-resistant acid phosphatase 5b; VAS, high-dose vitamin A supplement.

Mean serum TRACP 5b (osteoclast number), a measure of bone resorption, was lower in group 1 (high VA intake; VAS in past 30 d) than in groups 2 (high VA intake; VAS in past 3–6 mo) and 3 (low/adequate intake; VAS in past 3–6 mo) (Table 6). Mean serum PINP (osteoblast activity), a marker of bone formation, did not differ among groups.

**FIGURE 3:**
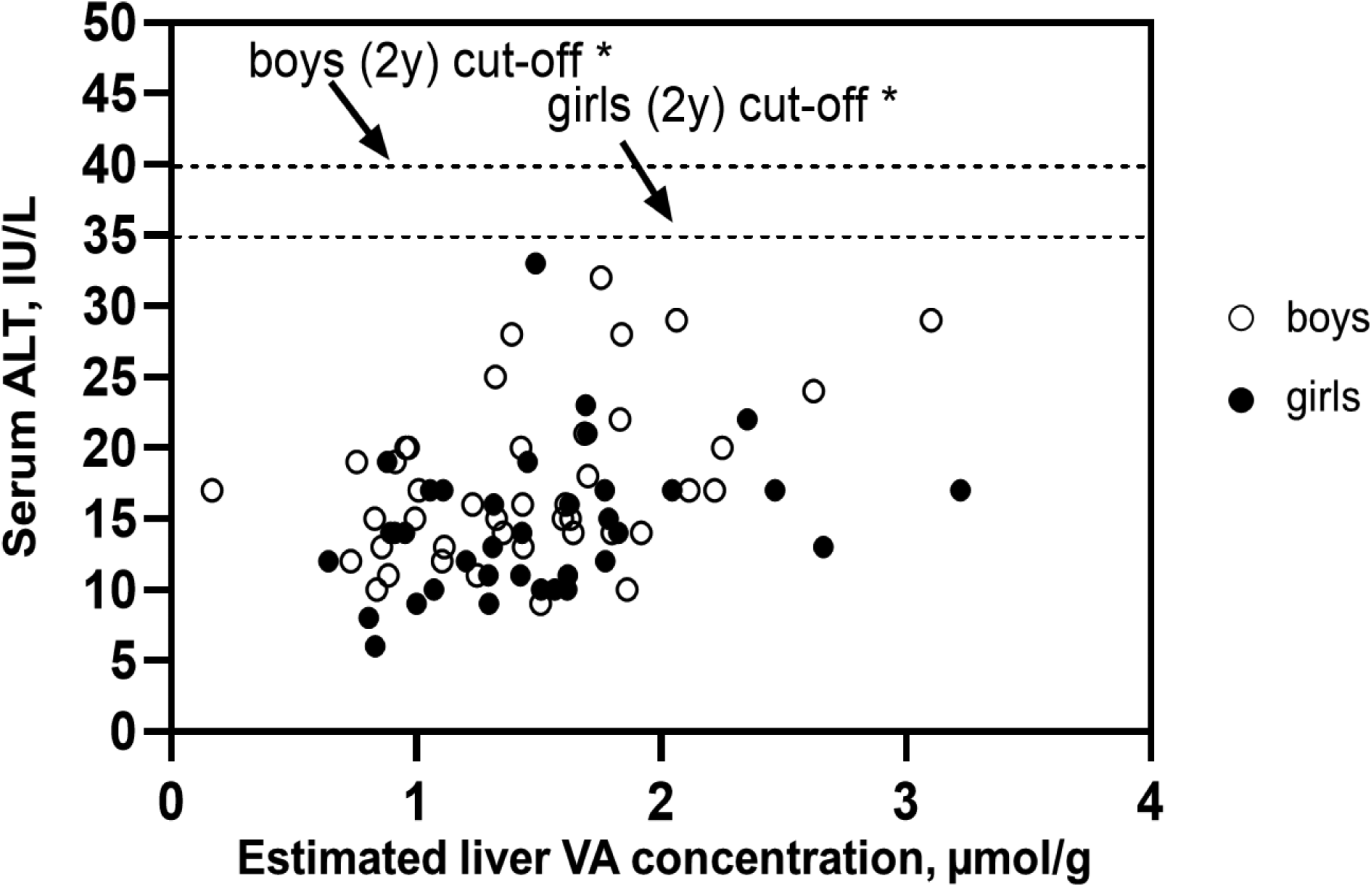
Serum ALT activity vs. estimated liver vitamin A concentration in relation to age- and sex-specific cutoff values for serum ALT activity. The dashed lines represent the age- and sex-specific cutoff values for serum ALT activity for children 2 y of age (42). *Boys: *r* = 0.429; *P* = 0.005; girls: *r* = 0.302; *P* = 0.0739; boys and girls combined: *r* = 0.351; *P* = 0.002. ALT, alanine aminotransferase; VA, vitamin A.

**FIGURE 4:**
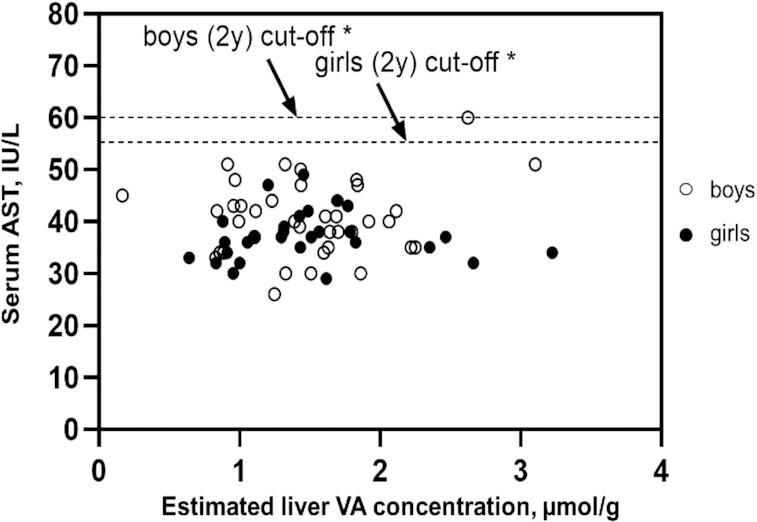
Serum AST activity vs. estimated liver vitamin A concentration in relation to age- and sex-specific cutoff values for serum AST activity. The dashed lines represent the age- and sex-specific cutoff values for serum AST activity for children 2 y of age (43). *Boys: *r* = 0.170; *P* = 0.307; girls: *r* = 0.007; *P* = 0.972; boys and girls combined: *r* = 0.105; *P* = 0.397). AST, aspartate aminotransferase; VA, vitamin A.

All children had serum ALT and AST concentrations within age- and sex-specific reference ranges [**Figures 3** and **4** (43, 44)], and the mean serum ALT and AST activities did not differ by group (Table 6). The mean serum AST:ALT ratio was higher in groups 1 and 2 (high-intake groups) than in group 3. None of the children had hepatitis A, B, or C. All children had serum albumin and total protein concentrations within the normal reference ranges [34–54 g/L (45) and 64–83 g/L (46), respectively], and mean values did not differ by group (**Supplemental Table 5**).

Associations between toxicity markers and estimated liver VA concentration and associations between toxicity markers and dietary VA intake are shown in **Supplemental Table 6** and **Supplemental Figures 3–5**. Serum ALT and plasma retinyl ester concentrations were positively associated with liver VA concentrations (*P* = 0.01); and the serum AST:ALT ratio was negatively associated with liver VA concentration (*P* = 0.02) (Supplemental Figures 3–5). Neither of the bone markers, nor serum albumin or serum total protein, were associated with liver VA concentration or dietary VA intake (Supplemental Table 6). Results of the analyses on the relations between biomarkers of VA toxicity and quintile of liver VA concentration were similar to analyses of individual observations (**Supplemental Figure 6**).

## Discussion

In this study of young Filipino children, most of whom had usual VA intake above the UL and who had received VAS either in the past 30 d or the past 3–6 mo, mean estimated liver VA concentrations were >1 µmol/g, which has been proposed as a threshold for high VA status (6), regardless of study group. As expected, children with higher reported VA intakes who received VAS in the past month had higher TBS, as assessed by RID, than those with lower VA intake who had not received VAS in the past 3 mo. However, we found no evidence of chronic VA toxicity based on serum markers of liver damage, bone turnover, or plasma concentrations of retinyl esters or 4-oxo-retinoic acid.

The mean retinol intake in children selected for the high intake groups was above the UL of 600 µg/d, and the prevalence of intakes above the UL was greater in the high-intake groups compared with the low/adequate-intake group, as expected. Mean energy intake was ∼22% higher than the estimated energy requirement of ∼713 kcal/d for children 1–2 y of age (based on kilocalories per kilogram per day) (47); however, this may be due to uncertainties around energy requirements for this population due to assumptions about physical activity levels. In any case, a potential overestimation of total intake of this magnitude would not change the conclusion that retinol intakes were generally above the UL. We had originally planned this study because of concerns about high exposure to VA through multiple supplementation and fortification programs. However, in this population, we found that the major dietary sources of VA included fortified milk powder (∼37%), low-dose, over-the-counter supplements (∼29%), and breast milk (∼16%), whereas public health programs contributed only a small proportion of total VA intake. Although wheat flour and cooking oil are fortified with VA, and >100 snack foods in the Philippines are fortified voluntarily by the food industry, children consumed small amounts of these foods, and thus they contributed little to total VA intake; also, while an MNP distribution program is in place, the product was not available in the community during the study.

TBS can be estimated in individuals using the RID method, which requires a single measurement of plasma retinol specific activity, and uses either an assumed or previously determined population-specific value for the coefficient *FaS* to predict TBS using the RID equation. The 28-d super-child study design that we used provides estimates of the geometric mean *FaS* coefficient for the group of children at each time point, which allows for TBS to be predicted at a selected time point using the corresponding *FaS* coefficient in the RID equation. In the present study, we determined TBS in individual children by using the RID method and the population-specific *FaS* coefficient at 4 d after dosing [see reference (22) for details on estimation of the *FaS* coefficient]. Reported values for the *FaS* coefficient among children at 4 d after isotope dosing are variable. In studies of theoretical children, *FaS* values of 1.51 and 1.58 were derived at 4 d for children with TBS of 29–1107 µmol and 700–2000 µmol, respectively (40, 41). Among Bangladeshi children (9–17 mo of age) with GM TBS of 199 µmol, the *FaS* coefficient at 4 d was 1.58; and for Guatemalan children (3–5 y of age) with GM TBS of 1068 µmol, it was 2.93 (22). For Mexican children (3–6 y of age) with GM TBS of 1097 µmol and Mexican children (17–35 mo of age) with GM TBS of 823 µmol, the *FaS* coefficients were 3.10 at 4 d and 2.9 at 3 d, respectively (48, 49). The *FaS* value that was derived for Filipino children at 4 d after dosing (2.44) was within the range of the reported values for young and preschool-age children (∼1.51–3.01). Because of the variability in *FaS* values in different populations, it is preferable to derive a population-specific value for *FaS*, when feasible.

The RID-predicted GM TBS of all individual children at 4 d after tracer administration was 518 µmol and is similar to the GM TBS of 533 µmol that was predicted by the compartmental model (22). GM TBS were higher in children with high intake who received VAS in the past 30 d than in children with low/adequate intake who received VAS in the past 3–6 mo. However, GM TBS did not differ between children in groups 2 (high VA intake, VAS 3–6 mo) and 3 (low/adequate intake; VAS in past 3–6 mo). The lack of difference between these 2 groups may be related to similar timing of receipt of VAS (past 3–6 mo) and adequate usual VA intake in both groups. Although mean usual VA intake was lower in group 3 than in the high-intake groups, intake was adequate in nearly all children. Because dietary VA is utilized in preference to mobilization of VA from stores to meet VA needs (50), children in group 3 may not have utilized their stores more than children in group 2, and that may explain why GM TBS in group 2 were only slightly higher than in group 3; this small difference between the groups would not be detectable with our sample size. The observation of different intakes but similar TBS between the 2 groups may also reflect inaccuracy in the dietary assessment methods or the relatively short time period over which dietary assessment was conducted (∼1 mo) relative to the time period over which children accrue VA stores.

In clinical VA toxicity, fasting serum retinyl ester concentrations are elevated when expressed per unit volume or as a percentage of total serum VA (51). It was not feasible for children in our study to be fasted before blood collection because of frequent feeding in this age group. Nevertheless, the median (25th, 75th percentile) plasma retinyl ester concentration (0.077 µmol/L; 0.051, 0.107) of our nonfasted children was far below the concentration of ≥0.380 µmol/L, which was associated with liver damage (based on elevated serum AST activity) in US elderly individuals (42) and much further below the serum retinyl ester concentration of 3.72 µmol/L of a 4-y-old girl with clinically diagnosed VA toxicity (51). Although plasma retinyl esters were positively associated with liver VA concentration, only 1 child in group 2 had an elevated plasma retinyl ester concentration; this may simply reflect recent VA intake.

Elevated plasma concentrations of 13-*cis*-4-oxo-retinoic acid have been observed in adults in response to consumption of large amounts of VA from supplements, fried liver, or liver paste (52–53) and may be a potential indicator of VA toxicity (54). Among our study children, there was no evidence of elevated 4-oxo-retinoic acid concentrations; the mean total (13-*cis* and all*-trans*) 4-oxo-retinoic acid concentration in the high-intake groups (6.4 nmol/L) was lower than the mean 13-*cis*-4-oxo retinoic acid concentration among fasted healthy Dutch women (8.1 nmol/L). Importantly, mean 4-oxo-retinoic acid concentrations did not differ by group, and 4-oxo-retinoic acid was not associated with liver VA concentration.

Animal studies show that chronic high VA intake can result in bone loss, although mixed results have been reported for humans (55). We measured serum markers of osteoclast number (TRACP 5b) and osteoblast activity (PINP) because these were negatively affected by excess VA intake in rats (56). Serum concentrations of TRACP 5b and PINP were lower in rats with high retinol intake compared with control rats. We observed lower mean serum TRACP 5b concentration in children in the high-intake group who had received VAS in the past 30 d, compared with the other 2 groups, but no difference in serum PINP concentrations among the 3 groups. These findings are similar to those reported in a recent study of 3–5-y-old South African children with adequate dietary VA intake whose serum concentrations of C-terminal telopeptide of type 1 collagen (CTX), a marker of bone resorption, decreased 4 wk after receipt of VAS (200,000 IU) compared with baseline values, but their serum concentrations of PINP remained unchanged (57). We used serum TRACP 5b concentration as a marker of bone resorption in our study, rather than serum CTX concentration, because recent food intake can affect serum CTX concentrations and our children were too young to be fasted overnight. In our study, neither serum TRACP 5b nor serum PINP concentrations were associated with liver VA concentration. Similarly, in the South African study, changes in serum CTX and PINP concentrations were not associated with estimated initial liver VA concentration or change in liver VA concentration, which was assessed using the RID method (57). Our results suggest that recent receipt of VAS, but not chronic dietary VA intake at or around the UL, may affect bone metabolism and should be investigated further.

It is important to point out that serum concentrations of bone markers at a single time point are difficult to interpret in young children because of high intra-individual variability during periods of high growth velocity (58). Serum TRACP 5b concentrations of our study children are similar to values reported for healthy Austrian children (*n* = 88) in the same age range (58); however, 23 (25%) Filipino children had values above the 97th percentile for the Austrian children. Serum PINP concentrations of our study children are similar to values reported for healthy Korean (*n* = 40) and UK (*n* = 11) children who were slightly younger (≤1 y of age) (59, 60).

Elevated serum AST and/or ALT activity and the serum AST:ALT ratio are indicators of liver damage; however, only elevated serum AST activity has been shown to be associated with high VA intake in US elderly individuals (42). All of our study children had serum AST and ALT activities below the age- and sex-specific cutoffs (43, 44) for hepatic transaminases. However, children in the high-intake groups had higher mean serum AST:ALT ratios than children in the low/adequate-intake group. It is unlikely that this is related to liver damage because their serum AST and ALT activities were within the normal range. Also, the serum AST:ALT ratios for our study children are similar to those reported for apparently healthy Mexican children 2–4 y of age (61). The associations between the hepatic transaminases and liver VA concentration were inconsistent. Serum AST activity was not associated with liver VA concentration, whereas serum ALT activity was positively associated with liver VA concentration and the serum AST:ALT ratio was negatively associated. Because of the inconsistency in the associations, and because all children had serum ALT and AST activities below the age- and sex-specific cutoffs, it is unlikely that the positive association between serum ALT concentration and liver VA concentration reflects liver damage. However, longer-term exposure to VA could potentially result in harm, and should be investigated further among older children to confirm no adverse consequences.

We did not observe a relation between usual dietary VA intake and TBS or estimated liver VA concentration among children who received VA >90 d prior to the RID study. Young children's feeding practices change rapidly in the first 2 y of life; therefore, dietary intake at 12–18 mo is not likely to represent usual intake since birth. Also, although the analysis excluded children who received VAS in the past 30 d, the relation between dietary VA intake and liver VA concentration may still be obscured by the contribution of VAS to TBS, given that children received VAS at different times, within the window of 3–6 mo before enrollment.

Finally, because the optimal sampling time for predicting TBS in children is not well defined, we compared RID-predicted TBS in individual children at 4 d and 16–28 d after dosing because recent studies have suggested that later sampling times (∼12–28 d) may provide more accurate results (40, 41). We found that the RID-predicted values for TBS were highly correlated at the early and later time points, and that the rank order was reasonably good. This is in agreement with results from a recent study among Mexican preschool-age children that showed that GM TBS values based on blood sampling at 4 d after dosing compared with 7–21 d after dosing did not differ [1096 µmol (IQR: 836–1492) vs. 1026 µmol (IQR: 768–1418)] (48). This is also in agreement with the study that was mentioned earlier that showed that RID-predicted TBS at 4 d provided reasonably accurate predictions of assigned TBS values for theoretical children (23). We also examined associations between TBS and toxicity markers by quintile in addition to the individual-level analyses and results were similar (Supplemental Figure 6, **Supplemental Discussion**). The concordant results suggest that the findings based on individual children are robust, even though RID-predicted TBS for individual children with a wide range of VA stores may be more accurate at ∼12–28 d after dosing (40).

In summary, we identified young Filipino children with usual retinol intakes above the UL of 600 µg/d, who received VAS, and whose estimated liver VA concentrations were ∼1–3 µmol/g. Although the vast majority of children had liver VA concentrations above the current cutoff of >1 µmol/g for “hypervitaminosis A,” they did not show any evidence of chronic VA toxicity. These results suggest that the liver cutoff value should be re-evaluated. In addition, due to limited data, the current UL for VA for children was extrapolated from that for adults (16). Thus, there is a need to establish the no-observed-adverse-effect level (NOAEL) for VA for young children, which could be informed by the data from the current study.

The strengths of the study are as follows: *1*) VA status of the children was assessed quantitatively using the RID equation with a population-specific value for the *FaS* coefficient, *2*) dietary VA intake was estimated using multiple assessment methods and the NCI method for estimating usual intake, and *3*) biomarkers of VA toxicity were measured. Limitations are that our sample size was small and estimated VA intake based on the screening tool resulted in incorrect group assignments for some children. Also, although children were exposed to VA-fortified oil and wheat flour, and voluntarily fortified snack foods, they did not consume large amounts of these foods, nor did they receive MNPs during the study period; thus, this study could not directly examine the potential for these public health programs to contribute to excessive VA intake and status. Additional studies should be conducted among children with high usual VA intake who are likely to consume MNPs and/or larger amounts of VA-fortified foods. More information is also needed on the relation between liver VA concentration and adverse physiological effects in humans to better estimate liver VA cutoff values for the “subtoxic” and “toxic” categories of status. Importantly, the results show that the RID method can be used to estimate liver VA concentration among young children at risk of high VA intake in low-income community settings, and should be used to monitor the safety of VA programs in countries where populations are exposed to multiple sources of VA.

## Supplementary Material

nzac115_Supplemental_File

## Data Availability

Data described in the manuscript are available at https://doi.org/10.25405/data.ncl.9771902.v1.

## Notes

Supported by the Bill & Melinda Gates Foundation (project number OPP1115464) and the International Atomic Energy Agency (technical contract project numbers 18964, 18835, and CRP E4.30.30).

Author disclosures: The authors report no conflicts of interest. The sources of support had no role in the design or implementation of the study or in data analysis or interpretation.

Supplemental Methods, Supplemental Results, Supplemental Tables 1–6, Supplemental Figures 1–6, and Supplemental Discussion are available from the “Supplementary data” link in the online posting of the article and from the same link in the online table of contents at https://academic.oup.com/cdn/

Abbreviations used: AGP, α_1_-glycoprotein; ALT, alanine amino transferase; AST, aspartate amino transferase; BRINDA, Biomarkers Reflecting Inflammation and Nutritional Determinants of Anemia; CRP, C-reactive protein; CTX, C-terminal telopeptide of type 1 collagen; EU, European Union; FNRI, Food and Nutrition Research Institute; GloVitAS, Global Vitamin A Safety Assessment; GM, geometric mean; LMIC, low- and middle-income country; MNP, micronutrient powder; NCI, National Cancer Institute; PINP, procollagen type 1 N-terminal propeptide; RAE, retinol activity equivalents; RBP, retinol-binding protein; RID, retinol isotope dilution; TBS, vitamin A total body stores; TRACP 5b, serum tartrate-resistant acid phosphatase 5b; UL, Tolerable Upper Intake Level; VA, vitamin A; VAS, high-dose vitamin A supplement(s).
