# Supplementary material for: Filipino Children with High Usual Vitamin A Intakes and Exposure to Multiple Sources of Vitamin A Have Elevated Total Body Stores of Vitamin A But Do Not Show Clear Evidence of Vitamin A Toxicity
Source: Curr Dev Nutr. 2022 Jul 25;6(8):nzac115. doi: 10.1093/cdn/nzac115 (PMC9429969; doi:10.1093/cdn/nzac115)
Supplement: nzac115_Supplemental_File [file nzac115_supplemental_file.docx]

**Supplemental Methods**

**Timing of super-child studies**

The kinetic studies for the 3 groups of children were conducted separately. Group 1 was enrolled in late October/early November 2016, ~1 mo after receipt of VAS during the campaign in ~Sept/Oct 2016. Group 2 was enrolled in September 2016, ~3-6 mo after receipt of VAS during the national campaign in ~March-May 2016. Group 3 was enrolled in February 2017, ~3-6 mo after receipt of VAS in Sept/Oct 2016. Children in group 2 did not receive VAS during the Sept/Oct 2016 campaign while they were enrolled in the study, so they were given VAS upon completion of study activities. Children in groups 1 and 3 completed the study before the next scheduled VAS campaigns and were not given VAS upon completion of the study because they would have been able to receive their scheduled doses at the time of the next national campaign.

**Estimation of vitamin A intake from breast milk**.

Casual milk samples were collected and breast milk vitamin A was expressed as nmol/g milk fat to adjust for variability in milk fat. Milk fat varies throughout the day and within a feeding episode; foremilk is low in fat whereas hind milk is high in fat (1). Because retinol is found in milk fat, the retinol content of casual milk samples was adjusted for milk fat. Vitamin A intake from breast milk was estimated using breast milk retinol concentration, expressed as µmol/L, and breast milk intake (L/d) (see below). To convert milk retinol from nmol/g milk fat to µmol/L, we used a milk fat value of 36.2 g/L; this was the mean milk fat value for full milk samples from an earlier pilot study in lactating Filipino women (2). In that study, milk collection was standardized to time of day, and all milk was expressed from a breast that had not been used to feed the infant for at least one hour. Samples of well-mixed fresh milk were taken for measurement of milk fat, in triplicate, using the creamatocrit method (3). The milk fat value of 36.2 g/L is similar to the value of 35.8 g/L from the literature (4).

**Estimation of average breast milk intake**.

An oral dose of 30 g of deuterium oxide (Cambridge Isotopes Laboratories, Inc., Tewksbury, MA, USA) was administered to mothers; saliva samples were collected from mothers and children at baseline and at 1, 2, 3, 4, 13 and 14 days after dosing (study days 6-20). Deuterium enrichment in saliva was measured by Fourier transform infrared spectroscopy at the Food and Nutrition Research Institute (Taguig City, The Philippines) (5). Breast milk intake (L/d) was determined by fitting a two-compartment steady state model of water flow to the deuterium enrichment data in mother-child pairs (5).

**Vitamin A content of fortified foods and daily supplements**

The vitamin A content of fortified wheat flour and fortified cooking oil was based on the median of measured values in samples of flour and oil collected from the participants’ households or local markets (see below). The vitamin A values for fortified powdered milk products and over-the-counter vitamin A-containing supplements were based on values on product labels; however, the values were confirmed by HPLC analysis of a subset of samples from the study area (see below). The vitamin A content of fortified snack foods was obtained from product labels. In a previous study, we confirmed that vitamin A values for those foods were as indicated on product labels (data not shown).

**Vitamin A content of wheat flour and vegetable oil**

Samples of wheat flour (~10 g) and vegetable oil (~10 mL) were collected from participants’ homes, when available, and from neighborhood markets. Flour was collected into Ziploc bags and wrapped in foil. Vegetable oil was collected into small plastic containers and wrapped in foil. Flour and oil samples were stored at -20°C until they were shipped to the University of California, Davis, on dry ice for analysis of vitamin A content using the iCheck Fluoro and iCheck Chroma (BioAnalyt, Teltow, Germany), for flour and oil, respectively.

**Vitamin A content of fortified powdered milk products and over-the-counter vitamin A-containing supplements**

Packets (n=5) of the two most commonly consumed brands of fortified powdered milk were purchased before the expiration date on the product label from markets in the participants’ neighborhoods. At the study lab, the packets (n=5) of each product were opened and mixed thoroughly. Composite samples (~10 g) of each mixed product were collected in Ziploc bags and wrapped in foil. The samples were stored at -20°C until they were shipped to the University of California, Davis, on dry ice for analysis of the vitamin A content.

Bottles of the most commonly consumed vitamin A-containing supplements (drops and syrup) were purchased before the expiration date on the product label from local markets (n=5 bottles of each of 5 products). At the study lab, the packages (n=5) of each product were opened and mixed together. Composite samples for each product (~10 mL) were placed in small amber bottles and hand-carried to the University of California, Davis, on ice packs for analysis of the vitamin A content. The fortified powdered milk products and the vitamin A-containing supplements were saponified and analyzed by HPLC, as described previously (6). The CV for retinol measurements for pooled plasma that was analyzed in triplicate with each batch of fortified milk and supplement samples was <5%.

**Supplemental Results**

**Vitamin A content of fortified wheat flour and vegetable oil**

For the 20 samples of wheat flour, the median (p25, p75) vitamin A concentration was 3.6 (1.7, 7.8) µg RE/g. Approximately 50% of samples had less than the target concentration of 4.5 µg RE/g (7, 8). For the 75 samples of vegetable oil, the median (p25, p75) vitamin A concentration was 17.2 (5.1, 24.6) µg RE/g. Approximately 44% of samples had less than the target concentration of 12 µg RE/g (9).

**Vitamin A content of breast milk**

Overall, the adjusted median (p25, p75) breast milk vitamin A concentration was 1.58 (1.22, 1.94) µmol/L (n=54) (based on the adjustment for milk fat described above). When milk retinol was expressed as nmol/g milk fat, the median (p25, p75) values were 43.7 (33.7, 53.6) nmol/g fat; breast milk intake results will be reported separately.

**Vitamin A content of over-the-counter supplements and fortified powdered milk products in relation to values on product labels**

The measured VA content of the most commonly consumed over-the-counter VA-containing supplements was 86-114% of the nutrient reference values for VA indicated on the product labels for 4 of 5 products and 57% for 1 product. The measured VA content of fortified powdered milk products was 99-104% of the values indicated on the product labels.

**Inflammation on the day of dosing and child morbidity**

We were not able to screen for elevated α1-acid glycoprotein (AGP) on the day of dosing; thus, it is possible that enrolled children were still experiencing some inflammation. The overall mean percent of days with reported symptoms of illness was low (cough or nasal discharge ~9.7% and 13.1%, respectively; fever or diarrhea ~ 2.4% and 1.8%, respectively) (**Supplemental Table 2**).

**Supplemental References**

1. Stoltzfus R, Underwood B. Breastmilk vitamin A as an indicator of the vitamin A status of women and infants. Bull World Health Org. 1995;73:703-11.

2. Engle-Stone R, Osei A, Dolly Reario M, Hall A, Arsenault J, Haselow N, Lietz G, Brown KH, Haskell MJ. Effect of short-term maternal supplementation with small amounts of vitamin A or beta-carotene on breast milk retinol concentrations among lactating Filipino women. Experimental Biology 2016. San Diego, CA, 2016.

3. Lucas A, Gibbs J, Lyster R. Creamatocrit: simple clinical technique for estimating fat concentration and energy value of human milk. Brit Med J. 1978;22:1019-20.

4. Dror DK, Allen LH. Retinol-to-fat ratio and retinol concentration in human milk show similar time trends and associations with maternal factors at the population level: A sysematic review and meta-analysis. Adv Nutr. 2018;9:332S-46S.

5. International Atomic Energy Agency (IAEA). Stable isotope technique to assess intake of human miilk in breastfed infants. IAEA, Vienna, Austria, 2010.

6. Turner T, Burri BJ. Rapid isocratic HPLC method and sample extraction procedures for measuring carotenoid, retinoid, and tocopherol concentrations in human blood and breast milk for intervention studies. Chromatographia. 2012;75:241-52.

7. Klemm RD, West KP, Jr., Palmer AC, Johnson Q, Randall P, Ranum P, Northrop-Clewes C. Vitamin A fortification of wheat flour: considerations and current recommendations. Food Nutr Bull. 2010 Mar;31:S47-61.

8. Allen L, de Benoist B, Dary O, Hurrell R, editors. Guidelines on food fortification with micronutrients. World Health Organization, Geneva, 2006.

9. Department of Health (DOH). Implementing Rules and Regulations for Mandatory Food Fortification. DOH, Manila, Philippines: 2000.

**Supplemental Tables**

| **SUPPLEMENTAL TABLE 1** Percent contribution of VA sources to total vitamin A intake | | | | | |
| --- | --- | --- | --- | --- | --- |
| VA source  N | | All  123 | Group 1  (high-intake + VAS in past 30 d)  47 | Group 2  (high-intake + VAS in past 3-6 mo)  39 | Group 3  (low/adequate-intake + VAS in past 3-6 mo)  37 |
| Complementary foods (including fortified powdered  milk), % | 55.8  52.2 (27.0, 89.6) | | 47.9  44.1 (20.8, 67.8) | 68.5  68.4 (45.8, 92.3) | 52.3  47.9 (25.3, 88.4) |
| Micronutrient  supplements^1^, % | | 28.5  28.6 (1.5, 47.8) | 40.5  43.6 (26.6, 56.2) | 24.2  15.4 (4.4, 45.8) | 17.8  11.1 (0, 31.4) |
| Breast milk, % | | 15.8  0 (0, 30.9) | 11.6  0 (0, 22.5) | 7.3  0 (0, 14.7) | 29.9  29.9 (0, 55.0) |

Values are mean and median (p 25, p 75) percentages of total vitamin A intake from each source of vitamin A, estimated using output of the indivint macro (see Methods). ^1^Includes daily over-the-counter supplements but not periodic VAS. VAS, high-dose vitamin A supplement.

| **SUPPLEMENTAL TABLE 2** Reported child morbidity during 28-d super-child study by study group | | | |
| --- | --- | --- | --- |
|  | Group 1  (high-intake + VAS in past  30 d) | Group 2  (high-intake + VAS in past 3-6 mo) | Group 3  (low/adequate-intake + VAS in past 3-6 mo) |
| N | 44 | 37 | 37 |
|  |  |  |  |
| Number of observed days, median (Q1, Q3) | 4.5 (4, 16) | 7 (4, 16) | 4 (4, 16) |
| % of individuals with any morbidity during observed period | 75.0 | 56.8 | 54.1 |
| Mean % of observed days with ^1:^ |  |  |  |
|  |  |  |  |
| Low appetite | 0 | 1.7 | 0 |
| Cough | 11.8 | 7.6 | 9.6 |
| Clear nasal discharge | 24.2^a^ | 11.7^b^ | 3.5^b^ |
| Purulent nasal discharge | 7.6^ab^ | 0.7^a^ | 13.8^b^ |
| Difficulty breathing | 0 | 0 | 0 |
| Fever | 3.4 | 1.3 | 2.5 |
| Diarrhea | 3.1 | 2.4 | 0 |
| Worms in stool | 0 | 2 | 0 |

Superscripts indicate Tukey-Kramer pairwise testing p<0.05 controlling for child age.^1^Symptoms of vomiting, nausea, urinary tract infection, blood or mucous in stool were not reported for any children. VAS, high-dose vitamin A supplement.

| **SUPPLEMENTAL TABLE 3** Inflammation and iron status indicators in children on study day 4 by study group | | | |
| --- | --- | --- | --- |
|  | Group 1  (high-intake + VAS in past 30 d) | Group 2  (high-intake + VAS in past  3-6 mo) | Group 3  (low/adequate-intake  + VAS in past 3-6 mo) |
| N | 43 | 35 | 35 |
|  |  |  |  |
| CRP, mg/L | 3.1 (8.5)^a^ | 1.2 (2.4)^a^ | 6.9 (16.5)^b^ |
| CRP >5 mg/L, % | 14.0 | 8.6 | 31.4 |
| AGP, g/L | 0.74 (0.42) | 0.72 (0.51) | 0.82 (0.53) |
| AGP >1 g/L, % | 16.3 | 20.0 | 25.7 |
| Any inflammation (CRP >5 mg/L and/or AGP >1 g/L), % | 25.6 | 22.9 | 40.0 |
| Ferritin, µg/L | 28.5 (20.1) | 29.6 (18.5) | 38.9 (28.6) |
| Ferritin <12 µg/L, % | 18.6 | 17.1 | 14.3 |
| Inflammation-corrected  ferritin, µg/L | 25.8 (32.3) | 26.6 (20.0) | 22.9 (13.8) |
| Inflammation-corrected  ferritin <12 µg/L, % | 32.6 | 22.9 | 20.0 |
| sTfR, mg/L | 7.5 (2.3) | 7.3 (1.5) | 7.8 (2.3) |
| sTfR >7.3 mg/L, % | 23.3 | 25.7 | 31.4 |
| Body iron stores (BIS), mg/kg | 2.5 (3.3) | 2.9 (2.9) | 3.5 (3.2) |
| BIS <0 mg/kg, % | 23.3 | 22.9 | 17.1 |

Values are mean (SD) and prevalences, %. Superscripts indicate Tukey-Kramer pairwise testing p<0.05. Serum ferritin was corrected for inflammation using the BRINDA method (31). AGP, α1-acid glycoprotein; BIS, body iron stores; CRP, C-reactive protein; sTfR, soluble transferring receptors; VAS, high-dose vitamin A supplement.

| **SUPPLEMENTAL TABLE 4**  Correlation matrix of vitamin A status indicators, fraction of isotope dose in plasma (FD_p_), plasma retinol specific activity/dose (SA_p_) and markers of inflammation on study day 4 | | | | | | |
| --- | --- | --- | --- | --- | --- | --- |
|  |  |  |  |  |  |  |
|  | TBS | Liver VA, µmol/g | Plasma retinol, µmol/L | Serum RBP, µmol/L | FD_p_ | SA_p_ |
|  |  |  |  |  |  |  |
| CRP, mg/L | 0.04  P=0.68  N=110 | 0.10  P=0.31  N=110 | -0.59  P<0.0001  N=112 | -0.50  P<0.0001  N=113 | -0.38  P<0.0001  N=112 | -0.04  P=0.66  N=112 |
| AGP, g/L | 0.04  P=.71  N=110 | 0.03  P=0.72  N=110 | -0.26  P=0.005  N=112 | -0.16  P=0.08  N=113 | -0.19  P=0.046  N=112 | -0.03  P=0.72  N=112 |
| Plasma retinol, µmol/L | -0.08  P=0.41  N=110 | -0.11  P=0.26  N=110 |  | 0.94  P<0.001  N=112 |  |  |
| Serum RBP, µmol/L | -0.09  P=0.36  N=110 | -0.12  P=0.21  N=110 |  |  |  |  |

Values are Spearman correlation coefficients, p-values, and numbers of observations compared. AGP, α1-acid glycoprotein; CRP, C-reactive protein; FD_p_, fraction of dose in plasma; RBP, retinol-binding protein; SA_p_, retinol specific activity in plasma; TBS, total body stores of vitamin A.

| **SUPPLEMENTAL TABLE 5** Serum albumin and total protein concentrations by study group | | | |
| --- | --- | --- | --- |
|  | Group 1  (high-intake + VAS in past 30 d) | Group 2  (high-intake + VAS in past 3-6 mo) | Group 3  (low/adequate-intake + VAS in past 3-6 mo) |
| N | 40 | 33 | 26 |
|  |  |  |  |
| Albumin, g/L | 45.3 (44.5, 46.1)^a^ | 47.5 (46.5, 48.5)^b^ | 45.8 (44.7, 47.0)^ab^ |
| Total protein, g/L | 67.3 (66.0, 68.5)^a^ | 71.9 (70.4, 73.5)^b^ | 69.9 (68.1, 71.7)^ab^ |

Values are mean (95% CI). Superscripts indicate Tukey-Kramer pairwise testing p<0.05 controlling for child age, serum CRP, serum AGP, length, BMI, child sex, and breastfeeding status. Sample size differs for group 2: Albumin N=32; Total protein N=33. VAS, high-dose vitamin A supplement.

| **SUPPLEMENTAL TABLE 6**  Regression estimates with 95% confidence intervals between biomarkers of vitamin A toxicity and protein status as predicted by liver vitamin A concentration or usual daily dietary vitamin A intake | | |
| --- | --- | --- |
| Liver vitamin A Usual vitamin A intake  (µmol/g) (µg RAE/d) | | |
| Serum TRACP 5b, U/L | -0.08 (-1.00, 0.84)  P=0.87  R^2^=0.13  N=90 | -0.28 (-0.97, 0.41)  P=0.42  R^2^=0.13  N=91 |
| Serum PINP, µg/L | -20.6 (-154.6, 113.4)  P=0.76  R^2^=0.16  N=102 | 9.90 (-96.2, 116.0)  P=0.85  R^2^=0.17  N=103 |
| Serum ALT, U/L | 3.18 (0.87, 5.48)  P=0.01  R^2^=0.32  N=75 | 0.16 (-1.51, 1.84)  P=0.85  R^2^=0.25  N=77 |
| Serum AST, U/L | 1.76 (-1.38, 4.89)  P=0.27  R^2^=0.13  N=65 | 0.17 (-2.13, 2.46)  P=0.88  R^2^=0.10  N=67 |
| Serum AST / ALT ratio | -0.40 (-0.72, -0.07)  P=0.02  R^2^=0.25  N=65 | 0.00 (-0.24, 0.25)  P=0.98  R^2^=0.19  N=67 |
| Plasma retinyl esters, µmol/L | 0.03 (0.01, 0.05)  P=0.01  R^2^=0.15  N=110 | 0.01 (-0.01, 0.03)  P=0.22  R^2^=0.11  N=113 |
| Plasma 4-oxo-retinoic acid, nmol/L | 0.36 (-1.26, 1.98)  P=0.66  R^2^=0.08  N=97 | -0.39 (-1.66, 0.88)  P=0.54  R^2^=0.07  N=99 |
|  |  |  |
| Serum albumin, g/L | 0.34 (-0.73, 1.41)  P=0.53  R^2^=0.10  N=95 | -0.22 (-1.03, 0.60)  P=0.60  R^2^=0.09  N=97 |
| Serum total protein, g/L | 0.52 (-1.25. 2.29)  P=0.56  R^2^=0.06  N=96 | -0.99 (-2.33, 0.34)  P=0.142  R^2^=0.08  N=98 |

Values are regression coefficients (95% CI) adjusted for child age, child sex, child weight, breastfeeding status, serum concentrations of CRP and AGP (i.e., for a one unit increase in the predictor indicated in the columns, the outcome indicated in the rows increases by the coefficient presented in the table). ALT, alanine aminotransferase; AST, aspartate aminotransferase; PINP, procollagen I intact N-terminal; RAE, retinol activity equivalent; TRACP 5b, tartrate resistant acid phosphatase 5b.

**Supplemental Figures**

**
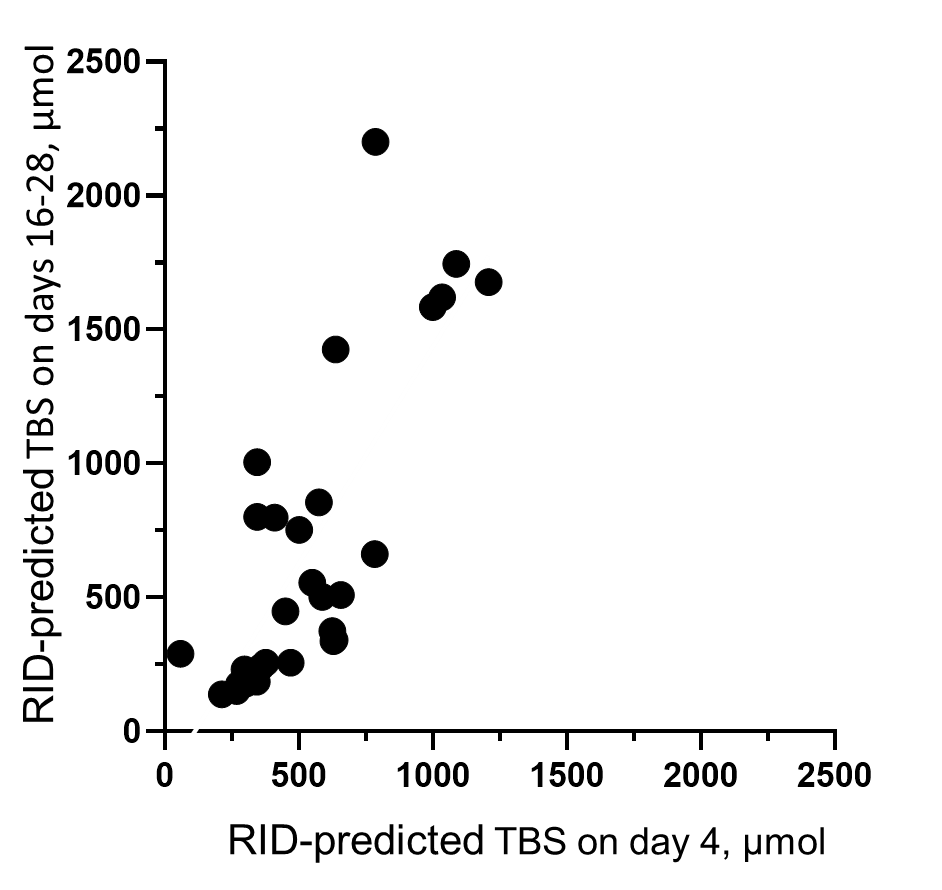
**

**SUPPLEMENTAL FIGURE 1** Correlation between RID-predicted TBS

at 4 d vs. 16-28 d after dosing (r = 0.78; P<0.0001; N=29). RID, retinol isotope

dilution; TBS, total body stores of vitamin A.

**
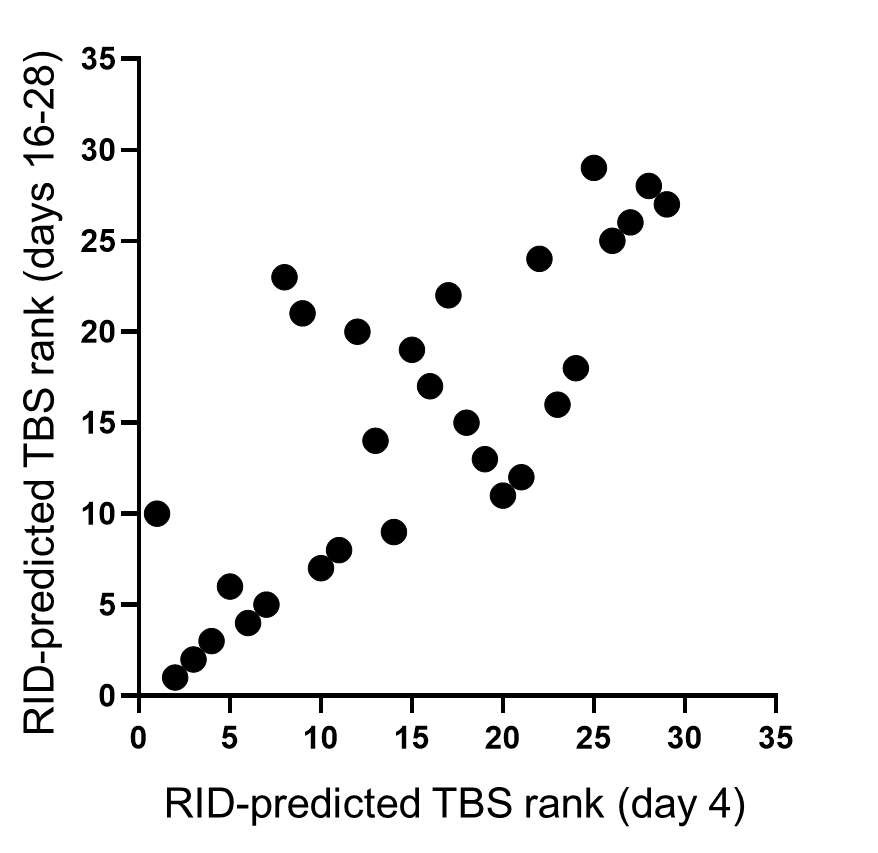
**

**SUPPLEMENTAL FIGURE 2** Rank-order values for RID-predicted

TBS at 4 d vs 16-28 d after dosing; (r= 0.77, P<0.0001, N=29). RID,

retinol isotope dilution; TBS, total body vitamin A stores.


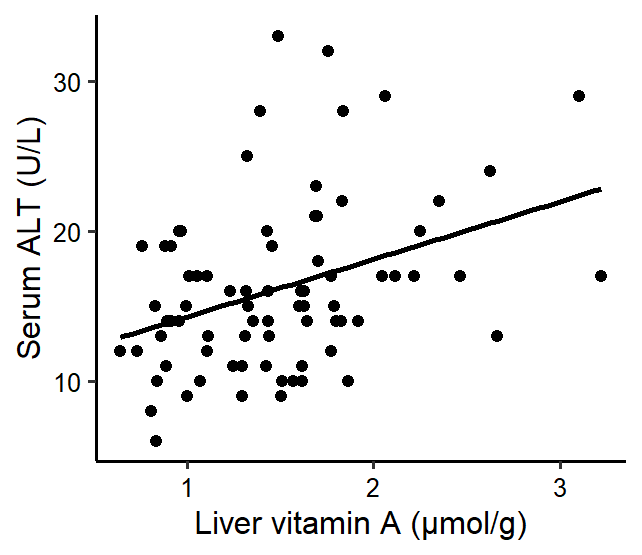


**SUPPLEMENTAL FIGURE 3** Scatter plot of liver vitamin A concentration

vs. serum ALT activity, N=75, R^2^=0.32. ALT, alanine aminotransferase.

.


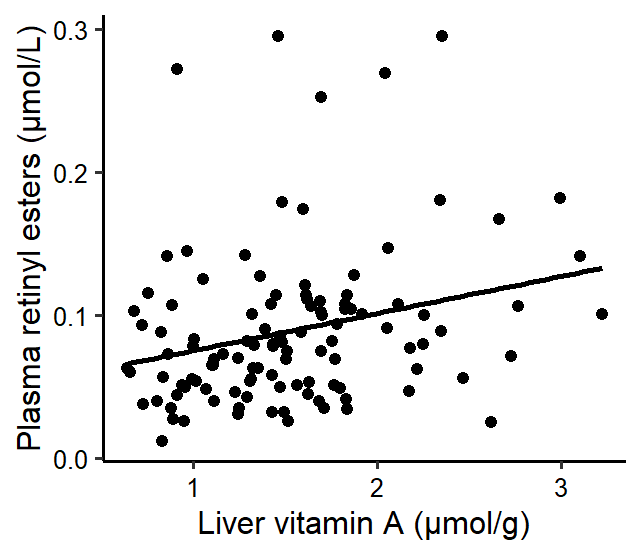


**SUPPLEMENTAL FIGURE 4** Scatter plot of liver vitamin A concentration

vs. plasma retinyl ester concentration, N=110, R^2^=0.15. Note: One outlier

value (0.646 µmol/L) was truncated to the next largest concentration (0.295 µmol/L)

for this analysis; a sensitivity analysis confirmed that the relationship remained similar

when the value for the outlier was included or excluded from the analysis.

.


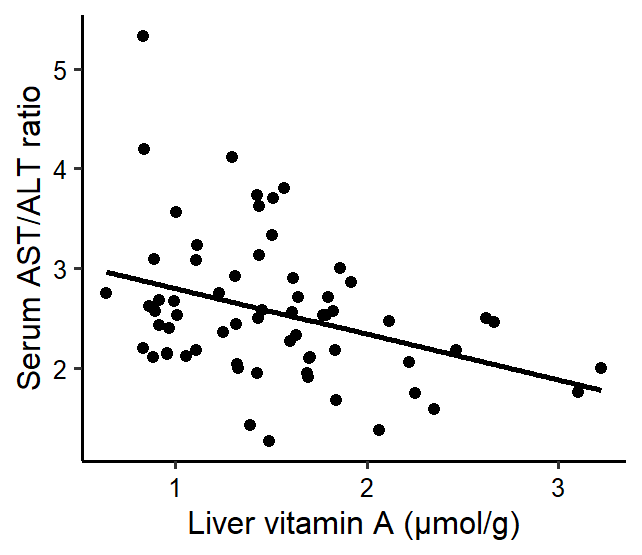


**SUPPLEMENTAL FIGURE 5** Scatter plot of liver vitamin A concentration

vs. serum AST/ALT ratio, N=65, R^2^=0.25. AST/ALT ratio,

aspartate aminotransferase/alanine aminotransferase ratio.

.

**SUPPLEMENTAL FIGURE 6** Biomarkers of VA toxicity vs. quintile of estimated liver VA concentration. Figures show box plots for each quintile (p25, median, p75). The black dots are individual data points. P-value for association in percentile regression models for figures A, B, and C were p=0.01, p=0.05 and p=0.07. ALT, alanine aminotransferase; AST/ALT ratio, aspartate aminotransferase/alanine aminotransferase ratio; VA, vitamin A.
